# Comparison of auditory sensations in patients who underwent cataract phacoemulsification surgery in the first and second eye

**DOI:** 10.1038/s41598-021-89594-6

**Published:** 2021-05-11

**Authors:** Joanna Konopińska, Dorota Ługowska, Zofia Mariak, Iwona Obuchowska

**Affiliations:** Department of Ophthalmology, Medical University of Bialystok, M. Sklodowska-Curie 24A, 15-276 Bialystok, Poland

**Keywords:** Diseases, Health care, Medical research

## Abstract

To compare subjective auditory sensations of patients during the first and second eye cataract surgeries. Consecutive patients who underwent phacoemulsification of the first eye (group I) and second eye (group II) completed questionnaires designed to evaluate their auditory sensations in the operating room including background music, sound of working equipment, staff conversations, and surgeon’s voice. This study included 124 patients in group I and 76 patients in group II. Patients most often heard nursing staff’s conversations (91.9% and 96%, respectively, p > 0.05), surgeon’s voice (87.9% and 86.8%, respectively, p > 0.05), and music (70.9% and 75%, respectively, p > 0.05). Music was the most pleasant experience (78.2% and 78.9%, respectively, p > 0.05). The sound of the working phacoemulsifier was the most undesirable sound (20.2% and 15.8%, respectively, p > 0.05). Patients in group II more often indicated that none of the sounds required elimination (69.7% and 52.6%, respectively, p = 0.013) or that staff conversations should be eliminated (13.2% and 3.1%, respectively, p = 0.005). The most desirable sounds during phacoemulsification include music and the surgeon’s voice regarding the procedure. The most unpleasant sound was that that of phacoemulsifier. The commonest sounds to be eliminated in groups I and II included those of equipment and staff conversations.

## Introduction

Cataract is the primary cause of blindness globally and is reversible with surgical procedures. Several efforts have been made to improve the procedure and make it as safe and less burdensome as possible for the patient. The surgical technique and method of anesthesia have been perfected, and much has been done to improve the comfort for the patient^1,2^. The introduction of topical anesthesia was one of the breakthroughs in cataract surgery as it allowed for the development of one-day surgery, significantly reduced the number of anesthesia-related complications, and significantly accelerated the rehabilitation of the eye after surgery^2–4^. In contrast, it should be noted that the patient remains conscious during the procedure and, therefore, is fully aware of the various stages of the procedure. This, in turn, may trigger anxiety and fear and consequently stimulate the sympathetic system, which manifests as tachycardia, increase in systemic blood pressure, hyperventilation, and even panic attacks^3,4^. Patients with cataracts are usually elderly with numerous comorbidities in whom the liberal or uncontrolled use of sedatives is not always possible^5^. Therefore, there is a need to identify the factors that increase the patient’s discomfort during the procedure and eliminate them to reduce the negative experiences. Such factors include auditory stimuli in the operating room, which may either alleviate anxiety or aggravate fear and sympathetic response.

Several studies have shown that listening to music before and during cataract surgery has positive effects in the patients. It has been demonstrated that music, as a nonpharmacologic intervention, immediately before the procedure significantly reduces the level of anxiety and fear^6–11^. Listening to music during the surgery may reduce pain perception^12^, reduce systemic blood pressure and heart rate, and decrease the need for sedative drugs^6,7,11,13^. Patients who listened to music during cataract surgery experienced less intraoperative anxiety if they heard no music or music of their choice as opposed to music selected by the surgeon^14,15^. However, few reports have highlighted that the patient’s comfort during phacoemulsification is influenced by other sounds from the environment. Sharma et al.^16^ evaluated the auditory perceptions during cataract surgery; however, their results did not provide any insights into perception of specific sounds and noises by the patients. No other studies have performed exact comparisons of auditory sensations between patients who underwent their first and second cataract surgery^16–23^.

In our research, we raised the question, if patients hear the conversations of the operating staff. We believe, that it is important for the surgeon to feel comfortable to know if the patient hear his voice and if he/she hears the commends. For this purpose we aimed to investigate patient feedback related to auditory sensations during cataract surgery, such as background music, phacoemulsification device sounds, staff conversations, and the voice of the operating surgeon. Furthermore, to precisely assess the importance of auditory stimuli and their effects on the patient’s feelings during the procedure, we compared the sensations between patients who underwent their first and second cataract surgery.

## Materials and methods

The study was performed in accordance with 1964 Declaration of Helsinki and its later amendments. The study was approved by the Bioethics Committee of the Medical University of Bialystok (R-I-002/398/2017), and the study protocol was registered on clinicaltrials.gov (NCT04327856).

This prospective, consecutive, observational study included 200 adult patients who underwent routine one-day phacoemulsification under topical anesthesia at the Department of Ophthalmology Medical University in Bialystok between January and March 2020. All patients provided written informed consent for the publication of the clinical data.

Patients who did not consent to participate in the study and those with difficulties in communication due to severe deafness, senile dementia, or mental disorders were excluded from this study. Patients who underwent complicated cataract surgery or multiple procedures during a single surgery were also excluded. All patients enrolled in the study were operated upon by the same surgeon (ZM).

Patients were divided into two groups. Group I included patients who underwent cataract surgery for the first time, and group II included those who underwent cataract surgery in their second eye.

The patients were instructed to complete a detailed questionnaire designed by the authors regarding the auditory sensations they experienced during phacoemulsification. The questionnaires were administered immediately after the surgery in the postoperative recovery room. The questionnaire consisted of closed-ended, single-answer, and multiple-choice questions. The survey included questions about the auditory sensations, such as background music, staff conversations, operating room noises, and surgeon’s instructions. The patients were offered assistance by nursing staff in reading, understanding, or completing the questionnaire. Each patient was interviewed for 15–20 min. The survey form is presented as supplementary information. Each patient was free to withdraw from the study at any time.

### Statistical analysis

We used statistical analyses reported in a previous study^18^. Survey data were evaluated using the statistical program R v3.5.4 (R Foundation for Statistical Computing, Vienna, Austria).

Answers to individual questions were mostly qualitative, and the qualitative variables were expressed as frequencies and percentages^18^. The normality of the quantitative variables was assessed using the Kolmogorov–Smirnov test. Given the absence of normal distributions, the quantitative variables were expressed as medians and median and IQR. Comparisons between patients undergoing either their first or second cataract surgeries were performed using the chi-squared test, Fisher’s exact test, and Mann–Whitney U test, as appropriate^18^. Certain questions were designed to include more than two possible answers. When the two groups demonstrated a statistically significant difference in the responses, a supplementary series of chi-squared post-hoc tests were performed to identify the precise answers that resulted in the difference^18^.

## Results

Group I included 124 (62%) patients with 74 (59.7%) women and 50 (40.3%) men. Group II included 76 (38%) patients with 45 (59.2%) women and 31 (40.8%) men. The mean age in group I and II was 72.7 ± 11.0 years (women: 73.2 ± 10.9 years; men: 71.8 ± 11.2 years) and 72.5 ± 11.0 years (women: 74.3 ± 9.9 years, men: 69.8 ± 12.2 years), respectively. There were no statistically significant differences in the age and sex distribution of the patients between the groups (p > 0.999 and p = 0.985, respectively). The groups also did not differ significantly in terms of education and place of residence.

Patients in groups I and II noticed sounds within the operating room (91.9% and 97.4%, respectively, p > 0.05). The characteristics of these sounds are summarized in Table 1.

**Table 1:** The auditory sensations experienced by patients during phacoemulsification.

	Group I		Group II		*p*
	*n*	%	*n*	%	
**Did you hear any sounds on the operating theater?**					
Yes	114	91.9%	74	97.4%	0.056
No	9	7%	2	2.1%	
Do not remember	1	1.1%	–	–	
**If yes, what kind of sounds?**					
Staff conversations	114	91.9%	73	96%	0.399
Surgeon comments and instructions	108	87.1%	66	86.8%	0.621
Music in the background	88	70.9%	57	75%	0.591
Noise of phacoemulsifier	64	51.6%	43	56.6%	0.070
**If you heard the phacoemulsifier noise, what kind of noise was that?**					
Rattling	37	29.8%	29	38.2%	0.327
Twitching	1	0.8%	2	2.6%	
Squeaking	2	1.6%	2	2.6%	
Hard to say	24	19.4%	10	13.2%	
**Did you listen intently to that sounds?**					
Yes	61	49.2%	40	52.6%	0.940
No	53	42.7%	34	44.7%	
**If you heard music, was it pleasant?**					
Yes	86	69.4%	56	73.7%	0.315
No	2	1.6%	1	1.3%	
**Do you think that the listening to the music during the surgery was beneficial?**					
Yes	102	82.3%	68	89.5%	0.341
No	21	16.9%	8	10.5%	
Do not know	1	0.8%	-	-	

Group 1: *N* = 124, Group 2: *N* = 76.

Regarding the most pleasant sounds, the patients could choose more than one answer. In terms of the sounds they would like to listen to during surgery in the future, a vast majority of patients answered with relaxation music (81.5% vs. 89.5% of patients in groups I and II, respectively, p > 0.05). The subjective assessments of sounds are summarized in Table 2.

**Table 2:** Subjective assessment of the sounds heard during the surgery.

	Group I		Group II		*p*
	*n*	%	*n*	%	
**Which sounds during the surgery you rate as a pleasure?**					
Music in the background	97	78.2%	60	78.9%	0.702
Staff conversations	5	4.0%	4	5.3%	
Surgeon informing about the course of surgery	83	66.9%	54	71.1%	
Noise of phacoemulsifier	-	-	-	-	
Do not know	5	4.0%	1	1.3%	
None	7	5.6%	2	2.6%	
**Which of the sounds were most unpleasant?**					
Staff conversations	6	4.8%	5	6.6%	
Noise of phacoemulsifier	25	20.2%	12	15.8%	0.226
Music in the background	13	10.5%	2	2.6%	
None	75	60.5%	53	69.7%	
Do not know	5	4.0%	4	5.2%	
**Which of the sounds during the surgery should be eliminated?**					
Staff conversations	4	3.2%	10	13.2%	0.005
Music in the background	2	1,6%	1	1.3%	0.008
Noise of phacoemulsifier	23	18.5%	7	9.2%	
None	65	52.4%^a^	53	69.7^a^	
Do not know	30	24.2^b^	5	6.6%^b^	

Group 1: *N* = 124, Group: *N* = 76.

^a,b^Statistically significant in post-hoc test (*a*: *p* = 0,013; *b*: *p* = 0,022), (*p* < 0,05).

Two (1.6%) patients in group I and 1 (1.3%) patient in group II reported that the background music of loud volume should be eliminated. Interestingly, as many as 93% patients in the entire study group (94.4% in group I and 92.1% in group II, p > 0.05) did not wish to have complete silence in the operating room during phacoemulsification.

Almost all patients in groups I and II understood the instructions provided to them by the surgeon (91.9% and 94.7%, respectively, p > 0.05). A majority of patients in groups I and II (81.5% and 84.2%, respectively, p > 0.05) reported that they would like to hear the surgeon’s words of encouragement during the operation along with constant updates. In contrast, the patients’ opinions were divided regarding being informed during the operation about the next stages as well as the current intraoperative difficulties and complications. Overall, 54% and 51.3% of patients in group I and II, respectively (p > 0.05) wished to receive information regarding the stages of surgery. The remaining patients were against receiving such information (46% vs. 47.4%, respectively, p > 0.05) or had no opinion (0% vs. 1.3%, respectively, p > 0.05). Additionally, 47.6% and 55.3% of those in groups I and II wished to be informed about complications during the surgery (p > 0.05). The remaining respondents answered the opposite (50.8% vs. 39.5%, respectively, p > 0.05) or did not state an opinion (1.6% vs. 5.3%, respectively, p > 0.05).

## Discussion

The aim of our study was to compare subjective auditory perceptions during cataract phacoemulsification in patients undergoing either their first or second cataract surgeries. Almost all patients experienced different auditory stimuli within the operating room. Most often, these were staff’s conversations, surgeon’s commands, soft music, and the sounds of the phacoemulsification apparatus less often. However, only half of the patients actively listened to the sounds they heard during the operation. Music was considered the most pleasant listening experience, followed by the voice of the surgeon who informed that the operation was going on as planned and would end soon. Most of the patients reported that relaxing background music was the most desirable sound during surgery. Similar answers were provided by patients of both groups.

Previous studies as well as our findings demonstrate that music positively influences the well-being of a patient in the operating theater^6–12,14,24^. Patients were more satisfied when they had the choice of whether to listen to relaxing music during surgery^15^. Some studies have demonstrated that music selected by the patients can be useful in reducing stress levels, even when it is not typically sedative. Patients who listened to music during cataract surgery reported less intraoperative anxiety if they heard no music or music that they had selected as opposed to music selected by the operating surgeon^14^.

In our study, a majority of the patients who underwent surgery for the first time and those who underwent it for the second time had a positive attitude toward music in the operating room. Only 3 of 200 patients found the music to be too loud. While Sharma et al.^16^ studied auditory perceptions during cataract surgery, their results did not state how the patients perceived specific sounds and noises. It is known that there are no differences in the perception of auditory sensations between the first and second cataract surgeries.

Our findings demonstrate shows that the most frequently heard sounds by the patients, apart from music, in the operating room included staff conversations and the surgeon’s voice. This may be because before cataract surgery, each patient is thoroughly educated on how to behave in the operating theater. One of the important elements of this education is to make the patient aware of the importance of cooperation in the operating theater between the patient and the medical staff and, particularly, the precise execution of the surgeon’s instructions. Over 80% of those in both groups wanted to hear the surgeon’s voice during the operation. The patients expected the surgeon’s words of encouragement, confirmation that the operation was progressing as planned, that nothing was wrong, and that the procedure would end soon. Only slightly more than 50% of the respondents (similar in both groups) wanted to be informed about the next stages of the operation on an ongoing basis, for example, “the cornea is cut”, “cloudy lens is removed”, or “artificial lens is being implanted”. A similar number of patients (47.6% and 55.3%, in groups I and II, respectively) wanted to be informed about the complications during the operation. Patients who did not wish to be informed about the course and complications of the operation during the operation did so because they found it too stressful and difficult to understand because of the specialized medical vocabulary. Therefore, the most important sound for the patient's comfort in the operating theater is the calm voice of the surgeon, which encourages and supports the patient. It is desirable to periodically reassure the patient that everything is progressing as planned without revealing any details.

The silence during phacoemulsification was reported to be not comfortable. Music or quiet conversation of the medical staff in the background act as “white noise” and soothe the patient who forgets about the tension and fear associated with the procedure and is often surprised that the operation is over.

Many of the patients from both groups were unable to identify the most unpleasant noise in the operating room that should be eliminated. People who underwent the second surgery more often recommended no sound that needed to be eliminated, which highlights that they understand that certain activities in the operating room are accompanied by certain noises that do not usually disturb the surgeries. The sound of the working equipment was considered the most unpleasant sound within the operating room in both groups, although it was only reported as such by 20% and 16% in group II and II, respectively. Nevertheless, the need to eliminate this noise was reported by 18.5% and 9% patients in group I and II, respectively, and the difference was statistically significant. This may suggest that people who were operated for the second eye have a greater knowledge of the surgery itself and, therefore, better understand that the phacoemulsifier must make such noises and that the sounds do not negatively affect the course of cataract extraction. Some patients believed that the noise that should be eliminated is that of staff conversations; additionally, this was the opinion significantly more often of patients who underwent the second operation (13% versus 3% in group I).

This study had some limitations. The study is based solely on self-reported questionnaires; therefore, all data are subjective. However, we assume that the patient’s comfort in the operating theater is extremely important, and if a given noise is subjectively evaluated as negative, then it should be addressed and eliminated. Another limitation may be the selection of patients. We compared people who underwent cataract surgery in the first eye with those who underwent surgery in the second eye. Therefore, the second group of patients could be less affected. However, both groups were comparable in terms of the demographic and clinical factors. We believe that such a comparison is valuable, especially, since the patients operated in the second eye had previously been operated in our clinic according to the same procedures. Future directions for such studies may cover the influences of other stimuli beside auditory ones, such as hand holding during the procedure^25,26^.

In conclusion, music received positive responses from patients who underwent cataract surgeries. One of the most desirable auditory sensations during phacoemulsification was the surgeon’s voice informing that the procedure is progressing as planned, without complications, and will end soon. The most unpleasant auditory experience was the sound of the phacoemulsifier. Many of the patients found none of the sounds in the operating room unpleasant. Patients undergoing cataract surgeries of the first and second eyes differed in terms of which noises should be eliminated. Only approximately half of the patients operated in each group wanted to be accurately informed during the surgery about its course and possible complications.

Based on our conclusions, we have formulated the following recommendations to improve the patient experience:

1. Introducing relaxing music in the operating theater according to a patient’s preferences.
2. Narration in surgeon’s calm voice about the course of the surgery.
3. The preparation procedures should be different according to first or second cataract surgery.
4. Patients operated for the second eye can be provided more comfort by reducing staff conversation.

## Supplementary Information

Supplementary Information.

## Data Availability

The datasets generated and/or analyzed during the current study are available from the corresponding authors on reasonable request; however, no information infringing on the privacy of the participants will be provided.
