# Supplementary material for: Comparison of auditory sensations in patients who underwent cataract phacoemulsification surgery in the first and second eye
Source: Sci Rep. 2021 May 11;11:10026. doi: 10.1038/s41598-021-89594-6 (PMC8113497; doi:10.1038/s41598-021-89594-6)
Supplement: Supplementary file 1 — Supplementary Information. [file 41598_2021_89594_MOESM1_ESM.pdf]

Comparison of auditory sensations in patients who underwent cataract phacoemulsification surgery in the first and second eye

Joanna Konopińska, Dorota Ługowska, Zofia Mariak, Iwona Obuchowska

Supplement

Patient Experience Survey

<u>Important!</u>

1) All answers should be marked with an "X" in the appropriate box after reading the question. Sometimes you will find an open answer question with some space for you to fill in the blanks. Please make sure you use clear handwriting.
2) Some questions are accompanied by comments to help you complete the survey correctly. They are marked in red italics, or diagonal letters, in brackets, e.g. (fill in the blanks). In case of any doubts - interviewer will try to explain them.
3) If you find this text impossible to read and therefore cannot provide your answers in writing, the interviewer will help in completing the questionnaire.

L.p.	Question	
1.	Did you hear any noises coming from the operating room?	☐ Yes<br>☐ No<br>☐ Not sure
2.	If you heard noises from the operating room, what was it?	☐ Background music<br>☐ Conversation between members of staff<br>☐ Instruction given by the surgeon<br>☐ Noise made by working equipment<br>☐ Other (please describe)……………………………………<br><br>*(you may choose more than one options)*
3.	Did you listen to these noises?	☐ Yes<br>☐ No<br>☐ Not sure
		If not, why ……………………………………
4.	If you heard music, did you enjoy it?	☐ Yes<br>☐ No<br>☐ Not sure
		If yes, why ……………………………………

5.	How would you describe the noise made by working equipment (phacoemulsifier)	☐ Rattling<br>☐ Howling<br>☐ Squeaking<br>☐ Vibration<br>☐ Knocking<br>☐ Did not hear anything<br>☐ Not sure<br>☐ Other:……………………. ……………………...…………...<br>*(you may choose more than one options)*
6.	What is the most pleasant noise from the operating room?	☐ Background music<br>☐ Conversation between members of staff<br>☐ Instruction given by the surgeon<br>☐ Noise made by working equipment<br>☐ None<br>☐ Other (please describe)…………………………………
7.	What noise in the operating room do you find the least pleasant?	☐ Background music<br>☐ Conversation between members of staff<br>☐ Instruction given by the surgeon<br>☐ Noise made by working equipment<br>☐ None<br>☐ Other (please describe)…………………………………
8.	Would you like complete silence in the room during the operation?	☐ Yes<br>☐ No<br>☐ Not sure

9.	Given the experience of the current surgery, what would you change in the operating room to be in the future?	…………………………………………………………………
10.	What noise in the operating room do you think should be eliminated?	☐ Background music<br>☐ Conversation between members of staff<br>☐ Instruction given by the surgeon<br>☐ Noise made by working equipment<br>☐ None<br>☐ Other (please describe)…………………………………
11.	Did you understand the instructions given in the operating theatre by the surgeon?	☐ Yes<br>☐ No<br>☐ Not sure
12.	Did you want to hear the operator's voice during the operation?	☐ Yes<br>☐ No<br>☐ Not sure<br><br>If yes, what you would like to hear most from him …………..
13.	Would you like to be updated on progress of the operation?	☐ Yes<br>☐ No<br>☐ Not sure

		If not, why ……………………………………
14.	Would you like to be informed about current complications during the operation?	☐ Yes<br>☐ No<br>☐ Not sure
		If not, why ……………………………………
